# Nigella sativa fixed and essential oil modulates glutathione redox enzymes in potassium bromate induced oxidative stress

**DOI:** 10.1186/s12906-015-0853-7

**Published:** 2015-09-18

**Authors:** Muhammad Tauseef Sultan, Masood Sadiq Butt, Roselina Karim, Waqas Ahmed, Ubedullah Kaka, Shakeel Ahmad, Saikat Dewanjee, Hawa ZE Jaafar, M. Zia-Ul-Haq

**Affiliations:** Institute of Food Science and Nutrition, Bahauddin Zakariya University, Multan, Pakistan; National Institute of Food Science and Technology, University of Agriculture, Faisalabad, Pakistan; Faculty of Food Science & Technology, University of Putra Malaysia, Serdang, Selangor, MY Malaysia; Faculty of Veterinary Medicines, University of Putra Malaysia, Serdang, Selangor Malaysia; Department of Agronomy, Bahauddin Zakariya University Multan, Multan, Pakistan; Advanced Pharmacognosy Research Laboratory, Department of Pharmaceutical Technology, Jadavpur University, Kolkata, 700032 India; Department of Crop Science, Faculty of Agriculture, University Putra Malaysia, Serdang, Selangor Malaysia; The Patent Office, Karachi, Pakistan

**Keywords:** *Nigella sativa*, Functional foods, Oxidative stress, Glutathione, Myeloperoxidase

## Abstract

**Background:**

*Nigella sativa* is an important component of several traditional herbal preparations in various countries. It finds its applications in improving overall health and boosting immunity. The current study evaluated the role of fixed and essential oil of *Nigella sativa* against potassium bromate induced oxidative stress with special reference to modulation of glutathione redox enzymes and myeloperoxidase.

**Methods:**

Animals; 30 rats (Sprague Dawley) were divided in three groups and oxidative stress was induced using mild dose of potassium bromate. The groups were on their respective diets (iso-caloric diets for a period of 56 days) i.e. control and two experimental diets containing *N. sativa* fixed (4 %) and essential (0.3 %) oils. The activities of enzymes involved in glutathione redox system and myeloperoxidase (MPO) were analyzed.

**Results:**

The experimental diets modulated the activities of enzymes i.e. glutathione-S-transferase (GST), glutathione reductase (GR) and glutathione peroxidase (GPx) positively. Indices of antioxidant status like tocopherols and glutathione were in linear relationship with that of GPx, GR and GST (*P* < 0.01). MPO activities were in negative correlation with GST (*P* < 0.01) but positive correlation with some other parameters.

**Conclusions:**

Our results indicated that both *Nigella sativa* fixed and essential oil are effective in improving the antioxidant indices against potassium bromate induced oxidative stress.

## Background

Since ages, communities relied on traditional medicines to prevent and cure various types of dietary health disorders. Scientists have realized now plant-based dietary interventions as the core heart epicenter of complimentary and alternative medicines. As the industrialization spun, the trends shifted towards reliance on pharmaceuticals. However, there are several societies across the globe who are more battened towards dietary therapeutic interventions [1, 2].

*Nigella sativa* L. (black cumin or black seeds) is good source of nutritionally essential components. Various cultures and civilizations utilize its seeds as herbal medicine to treat and prevent number of diseases [3]. Its health enhancing potentials are due to active ingredients (thymoquinone, carvacrol, thymol, cymene, t-anethole and 4-terpineol) that are mainly concentrated in fixed or essential oil [4]. Aforementioned bioactive components along with some others constituents possess notable antioxidant activity. *N. sativa* and its essential ingredient like thymoquinone are effective free radical scavengers in lipid peroxidation. Furthermore, they can also act as immune boosters and several research investigations have supported their anti-inflammatory and immuno-modulatory effects [5].

The production of free radicals is an integral part of body metabolism but imbalance results is oxidative stress. The excessive lipid peroxidation results in destruction of cellular membranes that could leads to cell death and degenerative disorders [6]. The modulation of lipid peroxidation through natural body defense mechanism involving hepatic enzymes is of considerable importance. Previously, scientists elaborated that the *N. sativa* essential oil tends to normalize the level of lipid peroxidase (LPX), lactate dehydrogenase (LDH), glutathione (GSH), superoxide dismutase (SOD), glutathione peroxidase (GPx) and catalase (CAT) enzymes [7]. Some other researchers outlined the importance of *N. sativa* as antioxidant that could be useful in pathological conditions related to free radicals. However, the combination and *in vivo* role of oxidative stress and antioxidants is still a matter of conjecture. The antioxidant potential seems inversely linked with oxidative damage, however, the interactions between antioxidant potential of *N. sativa* in oxidative stress conditions needs research interventions conducted comprehensively. In this context, we have tried to study the role of *Nigella sativa* fixed and essential oils (NSFO & NSEO) in improving the antioxidant status and attempted to explore their mechanisms and molecular targets. For the purpose, we measured the oxidative damage due to oxidative stress and determined the antioxidant potential of the body through measuring total antioxidant capacity (TAC), tocopherols, and glutathione contents. Later, we attempted to explore the mechanism and measured the glutathione redx enzymes expressions and myeleperoxidase activity.

## Methods

*Nigella sativa* seeds were procured from Barani Agricultural Research Institute, Chakwal (Voucher specimen No. Chk.Pk-926) and preserved in the same institute for future reference. The seeds were further identified by Dr. Qasim Ali, Department of Botany, University of Agriculture, Faisalabad. Chemical Reagents (analytical & HPLC grade) and standards were purchased from Sigma-Aldrich Tokyo, Japan and Merck KGaA, Darmstadt, Germany. The present research was conducted following the instructions of “Animal Care Committee, NIFSAT-Faisalabad Pakistan” (NIFSAT/ACC/07/16) and research plan was submitted to National Institute of Health (NIH), Islamabad for further approval of research. They provided infectious free Sprague dawley rats as per requirements of modified proposed research.

### Extraction of *Nigella sativa* fixed and essential oils

Following the standard procedures, the seeds of *Nigella sativa* were slurred with hexane in 1:6 ratio (Soxtech apparatus and rotary evaporator) to extract the fixed oil. *Nigella sativa* essential oil was extracted using locally assembled hydro-distillation apparatus. Earlier to this manuscript, the authors published the results pertaining to the nutritional composition of NSFO and NSEO and safety assessment in Food and Chemical Toxicology [8].

### Housing of rats

The National Institute of Health (NIH), Islamabad provided infectious free 30 Sprague Dawley rats that were further divided into three groups of ten rats each. The animals were maintained according to standard guidelines of Animal Institute of Nutrition (AIN), USA i.e. temperature 23 ± 2 °C, relative humidity 55 ± 5 %, and 12-h light–dark cycle. In the first week, the feed of the rats was basal diet in order to acclimatize them to new environment. After the acclimatization period, rats received their respective experimental diets for a period of 8 weeks (56 days) (Table 1).

**Table 1:** Composition of control and experimental diets

Diet constituents	D_1_	D_2_	D_3_
Casein (g)	20.0	20.0	20.0
Corn starch (g)	55.0	55.0	55.0
Cellulose	10.0	10.0	10.0
Corn oil (g)	10.0	6.0	10.0
*Nigella sativa* fixed oil	-	4.0	-
*Nigella sativa* essential oil	-	-	0.3
Mineral mixture (g)	4.0	4.0	4.0
Vitamin mixture (g)	1.0	1.0	1.0
Total diet weight (g)	100	100	100

D_1_ = Control diet; D_2_ = *Nigella sativa* seed fixed oil; D_3_ = *Nigella sativa* seed essential oil

In order to induce oxidative stress in rats, the peritoneal injection of potassium bromate @ 45 mg/Kg body weight dissolved in 0.05 M citrate buffer (pH 4.5) was employed. The analytical procedures carried out include feed & water intake (measured daily) and body weight (weekly basis). At 28 & 56 days of feeding trials, five rats from each group were decapitated for blood collection through neck and cardiac puncture [9]. The collected blood samples were analyzed for further assays and details are mentioned herein.

### Indices of oxidative damage

Indicators of lipid peroxidation were estimated including MDA level [10], total antioxidant capacity [11] and conjugated dienes according to protocol described by Corongiu and Milla [12]. For total antioxidant capacity, we added reagent-1 (xylenol orange, NaCl and glycerol) in 35 μL of collected serum and than added 11 μL of reagent-2 (ferrous ion and o-dianisidine, H_2_SO_4_ solution) and readings were taken at 560 nm and 800 nm. Briefly, MDA was measured with a colorimetric method using tetraethoxypropane as standard and units followed were nmol/mL. For the determination of conjugated dienes, serum was homogenized and centrifuged at 3000 g. The spectra obtained (232–247 nm) using hexane as standard solution was further used to estimate conjugated dienes and values were expressed in nmol/g of lipids.

### Indices of antioxidant status

Glutathione contents were determined following the protocols described by Beutler [13]. In the protein free supernatant, the colored product of GSH + DTNB was measured at 412 nm and expressed as nmol/mg protein. Level of serum α-tocopherol and γ-tocopherol were also measured following the procedures described by Xu and Godber [14] through HPLC. Briefly, the oil samples were slurred in hexane as a first step. A normal phase HPLC column (250 mm × 4.6 mm, 5.0 μm particle size) and mobile phase consisting of isooctane & ethyl acetate (96:4 v/v) was used for the purpose. Total run time and flow rate were 30 min and 1.0 mL/min, respectively. The detector was set at 290 nm excitation wavelength and 400 nm emission wavelengths. The column temperature was 35 °C. Similarly, the individual standards of isomers of tocopherols were run using the same pattern and curves were obtained and used for calibration in order to determine the amounts of tocopherols in *Nigella sativa* fixed oil.

### Superoxide dismutase and catalase enzymes assays

The antioxidant enzymes assays include the estimation of Superoxide dismutase (SOD) and catalase. The measurement of SOD activity was based on the reaction between ability of enzyme to inhibit cytochrome ‘c’ oxidation [15] and activity was expressed in IU/mg protein. The decomposition of hydrogen peroxide and measurement of its by-products were mainly used for the estimation of catalase (CAT) activity. The units remained the same as IU/mg protein and 1 IU was equivalent to 1 μmol H_2_O_2_ consumed per mg protein per minute [16].

### Glutathione redox enzymes assays

Glutathione transferase (GST) action was measure using the commercial kits provided by Bioassay Internationals. The reaction include the rate of formation of conjugate between GSH and 1-chloro-2,4- dinitrobenzene [13] and measurement unit used IU/mg protein. The 1 IU is equivalent to 1 μmol of conjugate formed/min/mg protein. Glutathione peroxidase (GPx) activity was estimated using tertiary butyl hydroperoxide (tbHP) as substrate [17] and the activity was expressed in IU/mg protein. The 1 IU is equivalent to one nmol of NADPH oxidized/mg protein in one minute. Glutathione reductase (GR) activity was assayed by estimating the oxidation of NADPH. Glutathione reductase (GR) activity was assayed at 37 °C and 340 nm by estimating the oxidation of NADPH. Hepatic enzymes like GST, GPx and GR was determined as described by Paglia and Valentine [18].

### Myeloperoxidase, xanthine oxidase, and nitric oxide

In this study, we used spectrophotometer to estimate the activity of tissue associated myeloperoxidase (MPO) following the procedures laid down by Hillegas et al. [19]. For the purpose, single unit of enzyme activity (IU/mg of proteins) was defined as the amount of the MPO present that caused a change in absorbance measured at 460 nm during the reaction time of three minutes. The indices like xanthine oxidase and nitric oxide are also of significance importance as far as immunopotentiating properties are concerned. We measured the xanthine oxidase using the using diagnostic kits from Cayman Chemicals.

### Statistical analysis

Statistical package i.e. Cohort V-6.1 (Co-Stat Statistical Software, 2003) was used for data analysis. Briefly, values presented in Tables are means ± standard deviation. In order to check the level of significance, analysis of variance (ANOVA) technique was applied. The diets (factor A), intervals (factor B) and their interaction (A × B) were used as source of variations (SOV). Duncan’s multiple range test (DMRt) further clarified the effects of diets and intervals in a comprehensive manner.

## Results

The onset of 21^st^ century witnessed the coinage of new terms like functional, nutraceutical and pharma foods. The awareness among the masses regarding the diet-health linkages improved significantly that further led scientists to conduct research studies. The present research intervention focused on exploring the role of *Nigella sativa* fixed oil (NSFO) and essential oil (NSEO) to improve antioxidant status and modulation of glutathione redox enzymes. The results pertaining to the different parameters presented herein.

### Indices of oxidative damage

Diets affected total antioxidant capacity (TAC), serum MDA and conjugated dienes levels significantly, whilst non-momentous impact of study intervals was observed (Table 2). The maximum antioxidant capacity of 0.62 ± 0.113 IU/mL was observed in groups of rats fed on D_3_ (*N. sativa* essential oil) followed by 0.52 ± .0044 IU/mL in D_2_ (*N. sativa* fixed oil), while minimum capacity of 0.40 ± 0.045 IU/mL was recorded for control group. However, total antioxidant capacity decreased in control group during the course of study from 0.49 ± 0.031 to 0.34 ± 0.021 IU/mL whereas increasing tendency from 0.44 ± 0.028 to 0.59 ± 0.035 and 0.43 ± 0.017 to 0.82 ± 0.035 IU/mL was observed in fixed and essential oils groups, respectively. The maximum MDA level 19.04 ± 1.54 nmol/g was recorded in D_1_ (control) followed by 13.97 ± 0.93 in D_2_ (*N. sativa* fixed oil), while lowest serum MDA level of 10.53 ± 2.47 nmol/g was recorded in D_3_ (*N. sativa* essential oil). During the course of study, progressive increase in serum MDA level was observed in control group from 16.90 ± 1.08 to 22.02 ± 1.37 nmol/g. Diet containing essential oil showed marked decrease in serum MDA level from 15.42 ± 0.61 to 7.46 ± 0.32 nmol/g during entire study. Likewise, progressive decrease in MDA level was also observed in fixed oil group from 15.60 ± 1.01 to 12.39 ± 0.73 nmol/g during 8 weeks study. Conjugated dienes varied differently among diets; group of rats fed on *N. sativa* fixed and essential oils showed significantly lower values i.e. 5.05 ± 0.253 and 4.49 ± 0.538 nmol/g, respectively as compared to maximum 6.22 ± 0.413 nmol/g in control group. Furthermore, conjugated dienes increased from 5.60 ± 0.359 to 7.00 ± 0.435 nmol/g in D_1_ group, while there observed decreasing trend from 5.48 ± 0.353 to 4.60 ± 0.270 and 5.26 ± 0.208 to 3.45 ± 0.148 nmo/g in D_2_ and D_3_ groups, respectively.

**Table 2:** Indices of antioxidants damage in oxidative stressed rats

Parameters	Diets	Study intervals (days)		
		0	28	56
Total antioxidants capacity (TAC) (IU/mL)	D_1_	0.49 ± 0.031cd	0.38 ± 0.027ef	0.34 ± 0.021f
	D_2_	0.44 ± 0.028de	0.54 ± 0.025bc	0.59 ± 0.035b
	D_3_	0.43 ± 0.017de	0.62 ± 0.030b	0.82 ± 0.035a
MDA (nmol/g)	D_1_	16.90 ± 1.08c	18.21 ± 1.31b	22.02 ± 1.37a
	D_2_	15.60 ± 1.01c	13.92 ± 0.65d	12.39 ± 0.73d
	D_3_	15.42 ± 0.61c	8.70 ± 0.42e	7.46 ± 0.32e
Conjugated dienes (CD) (nmol/g)	D_1_	5.60 ± 0.359bc	6.05 ± 0.436b	7.00 ± 0.435a
	D_2_	5.48 ± 0.353bc	5.07 ± 0.237cd	4.60 ± 0.270e
	D_3_	5.26 ± 0.208cd	4.76 ± 0.228de	3.45 ± 0.148f

Means sharing same letters in a column/row do not differ significantly at *P* < 0.05

### Indices of antioxidant status

It is apparent from Table 3 that glutathione, α- and β-tocopherols contents altered significantly as a function of experimental diets, study intervals and their interaction. Diets containing *N. sativa* fixed and essential oils showed promising improvements in glutathione contents i.e. increased from 29.09 ± 1.87 to 30.34 ± 1.78 and 26.76 ± 1.06 to 32.60 ± 1.39 mg/L, respectively, during the whole study duration. However, there observed progressive decrease in glutathione contents in D_1_ (control) from 27.70 ± 1.78 to 20.71 ± 1.29 mg/L. Similarly, α-Tocopherol contents decreased in control group significantly from 74.16 ± 4.75 to 52.35 ± 3.26 ng/mL. However, diets containing fixed and essential oils improved the tocopherols contents momentously from 70.51 ± 2.78 to 80.29 ± 3.43 and 70.97 ± 4.57 to 76.59 ± 4.50 ng/mL, respectively. Likewise, γ-Tocopherol contents decreased from 15.12 ± 0.97 to 12.01 ± 0.75 ng/mL in D_1_, while increased from 14.25 ± 0.56 to 19.06 ± 0.82 and 14.12 ± 0.91 to 16.91 ± 0.99 ng/mL in D_2_ and D_3_ groups, respectively.

**Table 3:** Indices of antioxidant status in oxidative stressed rats

Parameters	Diets	Study intervals (days)		
		0	28	56
Total Glutathione (mg/L)	D_1_	27.70 ± 1.78cd	23.24 ± 1.68e	20.71 ± 1.29f
	D_2_	29.09 ± 1.87c	28.03 ± 1.31cd	30.34 ± 1.78bc
	D_3_	26.76 ± 1.06de	30.71 ± 1.47b	32.60 ± 1.39a
Reduced Glutathione (mg/L)	D_1_	21.67 ± 1.25	17.80 ± 0.58	15.46 ± 0.78
	D_2_	23.19 ± 0.97	22.96 ± 0.77	24.93 ± 1.10
	D_3_	20.75 ± 0.79	25.33 ± 1.23	27.49 ± 1.22
α-Tocopherol (ng/mL)	D_1_	74.16 ± 4.75bc	61.36 ± 4.42d	52.35 ± 3.26e
	D_2_	70.51 ± 2.78c	78.55 ± 3.76a	80.29 ± 3.43a
	D_3_	70.97 ± 4.57c	74.92 ± 3.50bc	76.59 ± 4.50ab
γ-Tocopherol (ng/mL)	D_1_	15.12 ± 0.97cd	14.97 ± 1.08cd	12.01 ± 0.75e
	D_2_	14.25 ± 0.56d	16.58 ± 0.79b	19.06 ± 0.82a
	D_3_	14.12 ± 0.91d	15.66 ± 0.73bc	16.91 ± 0.99b

D_1_ = Control diet; D_2_ = *N. sativa* seed fixed oil; D_3_ = *N. sativa* seed essential oil

Means sharing same letters in a column/row do not differ significantly at *P* < 0.05

### Superoxide dismutase and catalase enzymes assays

The antioxidant enzymes including superoxide dismutase (SOD) and catalase (CAT) were significantly affected as a function of diets, study intervals and their interaction (Table 4). It is obvious from the results that SOD activity increased during the oxidative stress conditions that was more pronounced in control i.e. 17.66 ± 1.13 to 28.55 ± 1.78 IU/mg protein. The fixed and essential oils restored their activities from 18.39 ± 1.18 to 17.32 ± 1.02 and 18.21 ± 0.72 to 16.12 ± 0.69 IU/mg protein, respectively. The activity of catalase increased significantly in D_1_ (control) from 6.04 ± 0.376 to 7.19 ± 0.461 IU/mg protein, while the declining tendency from 8.10 ± 0.476 to 6.96 ± 0.448 and 10.83 ± 0.419 to 6.39 ± 0.252 IU/mg protein was observed in D_2_ (*N. sativa* fixed oil) and D_3_ (*N. sativa* essential oil) groups, respectively (Table 4).

**Table 4:** Hepatic antioxidants enzymes in oxidative stressed rats

Parameters	Diets	Study intervals (days)		
		0	28	56
SOD (IU/mg protein)	D_1_	17.66 ± 1.13c	24.94 ± 0.61b	28.55 ± 1.78a
	D_2_	18.39 ± 1.18c	18.09 ± 0.84c	17.32 ± 1.02cd
	D_3_	18.21 ± 0.72c	16.42 ± 0.79de	16.12 ± 0.69e
Catalase (IU/mg protein)	D_1_	6.04 ± 0.376e	6.40 ± 0.462e	7.19 ± 0.461de
	D_2_	8.10 ± 0.476bc	7.85 ± 0.367cd	6.96 ± 0.448de
	D_3_	10.83 ± 0.419a	8.83 ± 0.423b	6.39 ± 0.252e
Glutathione peroxidase (IU/mg protein)	D_1_	65.60 ± 4.206cd	60.72 ± 4.379d	58.92 ± 3.665d
	D_2_	58.01 ± 3.737d	67.73 ± 3.162c	72.68 ± 4.269bc
	D_3_	56.80 ± 2.243d	76.54 ± 3.619ab	80.79 ± 3.456a
Glutathione reductase (IU/mg protein)	D_1_	16.35 ± 1.048c	15.11 ± 1.089cd	14.70 ± 0.914d
	D_2_	14.48 ± 0.932d	24.38 ± 1.138b	25.35 ± 1.489b
	D_3_	14.62 ± 0.560d	26.55 ± 1.271a	27.60 ± 1.621a
Glutathione transferase (IU/mg protein)	D_1_	0.53 ± 0.034de	0.48 ± 0.035ef	0.40 ± 0.025f
	D_2_	0.43 ± 0.028f	0.56 ± 0.026d	0.61 ± 0.036c
	D_3_	0.42 ± 0.017f	0.67 ± 0.032b	0.73 ± 0.031a

D_1_ = Control diet; D_2_ = *N. sativa* seed fixed oil; D_3_ = *N. sativa* seed essential oil

Means sharing same letters in a column/row do not differ significantly at *P* < 0.05

### Glutathione redox enzymes assays

The experimental diets affected the enzyme expressions of glutathione peroxidase (GPx), glutathione reductase (GR) and glutathione transferase (GST) significantly (Table 4). However, GPx, GR and GST followed the opposite trend to SOD and catalase i.e. decreased significantly in control group from 65.60 ± 4.206 to 58.92 ± 3.665, 16.35 ± 1.048 to 14.70 ± 0.914 and 0.53 ± 0.034 to 0.40 ± 0.025 IU/mg protein, respectively (Table 4). Diets containing fixed and essential oils enhanced the expression of glutathione peroxidase significantly i.e. 58.01 ± 3.737 to 72.68 ± 4.269 and 56.80 ± 2.243 to 80.79 ± 3.456 IU/mg protein, respectively. Moreover, glutathione reductase increased from 14.48 ± 0.932 to 25.35 ± 1.489 and 14.62 ± 0.560 to 27.60 ± 1.621 IU/mg protein. Likewise, glutathione transferase contents increased from 0.43 ± 0.028 to 0.61 ± 0.036 and 0.42 ± 0.017 to 0.73 ± 0.031 IU/mg protein, respectively.

### Immuno-potentiating perspective

The experimental diets affected xanthine oxidase, nitric oxide and myeloperoxidase significantly (Table 5). The maximum xanthine oxidase activity (27.28 ± 1.23 IU/mg protein) was observed in D_1_ (control) followed by 19.12 ± 1.83 IU/mg protein in D_2_ (*N. sativa* fixed oil), while the least activity of 15.19 ± 2.96 IU/mg protein was recorded in D_3_ (*N. sativa* essential oil). Xanthine oxidase activity decreased significantly in D_2_ and D_3_ groups from 22.77 ± 1.47 to 17.44 ± 1.02 and 21.07 ± 0.83 to 10.91 ± 0.47 IU/mg protein, respectively during 8 weeks. The nitric oxide was maximum (36.40 ± 2.25 nmol/dL) in control followed by 32.38 ± 1.30 nmol/dL in D_2,_ while the minimum contents of 28.83 ± 2.46 nmol/dL were noted in D_3_ group. For same trait, increasing tendency was observed in control from 32.92 ± 2.11 to 40.62 ± 2.53 nmol/dL during 56 days of study. In contrast, D_2_ and D_3_ groups reduced the stress conditions by decreasing the nitric oxide level from 34.77 ± 2.24 to 30.31 ± 1.78 and 33.28 ± 1.31 to 24.80 ± 1.06 nmol/dL, correspondingly. The activity of MPO increased in D_1_ with the passage of time i.e. 18.91 ± 1.212 to 22.82 ± 1.420 IU/mg protein during study duration. In contrary, D_2_ (*N. sativa* fixed oil) and D_3_ (*N. sativa* essential oil) groups showed decrease in MPO activity from 19.43 ± 1.252 to 16.07 ± 0.944 and 20.62 ± 0.814 to 13.68 ± 0.585 IU/mg protein, respectively (Table 5).

**Table 5:** Indices of immune system in oxidative stressed rats

Parameters	Diets	Study intervals (days)		
		0	28	56
Xanthine oxidase (IU/mg protein)	D_1_	24.98 ± 1.60bc	27.69 ± 2.00ab	29.17 ± 1.81a
	D_2_	22.77 ± 1.47c	17.15 ± 0.80d	17.44 ± 1.02d
	D_3_	21.07 ± 0.83c	14.90 ± 0.71e	10.91 ± 0.47e
Nitric oxide (nmol/dL)	D_1_	32.92 ± 2.11d	35.66 ± 2.57b	40.62 ± 2.53a
	D_2_	34.77 ± 2.24bc	32.05 ± 1.50d	30.31 ± 1.78ef
	D_3_	33.28 ± 1.31cd	28.40 ± 1.36f	24.80 ± 1.06g
MPO (IU/mg protein)	D_1_	18.91 ± 1.212c	20.05 ± 1.446b	22.82 ± 1.420a
	D_2_	19.43 ± 1.252bc	18.93 ± 0.884c	16.07 ± 0.944d
	D_3_	20.62 ± 0.814ab	16.14 ± 0.772d	13.68 ± 0.585e

D_1_ = Control diet; D_2_ = *N. sativa* seed fixed oil; D_3_ = *N. sativa* seed essential oil

Means sharing same letters in a column/row do not differ significantly at *P* < 0.05

## Discussion

The diet and health linkages are well defined and many research studies carried out in animal and humans provided evidences that dietary modules are best strategy in preventing many ailments e.g. cardiovascular disorders and diabetes mellitus [4]. *Nigella sativa* is famous in Asian countries owing to its potential role in prevention of different ailments. In this regard, Sultan et al. [8] carried out the safety assessment of *Nigella sativa* fixed and essential oil (NSFO & NSEO). In the present research, further efforts were made to study the effects of NSFO and NSEO on hepatic enzymes, antioxidant status and immunopotentiating potential of *Nigella sativa* in oxidative stressed Sprague dawley rats. The protocols followed in the present research were based on the studies conducted by scientists [20, 21].

In the present study, we fed the groups of rats on experimental diets containing *N. sativa* fixed and essential oils along with control for a period of 8 weeks. The addition of *N. sativa* fixed and essential oil increased feed and water intakes as compared to control group that certainly be due to the remedial action of experimental diets thus resulting in restoration of normal metabolism and body homeostasis [22–24]. Oxidation and its complexities results in uncontrolled free radicals production that become lethal leading to oxidative stress. In this regard, these reactive oxygen species (ROS) overcomes the antioxidant capacity of the target cells and tissues. In stress-induced conditions, supplementation of antioxidants may evoke certain protective roles in the body against various health discrepancies [25]. In the instant study, total antioxidant capacity (TAC) of the body decreased significantly in control group, while values for serum MDA and conjugated dienes levels increased indicating more oxidative damage. However, diets containing *N. sativa* fixed and essential oils assuaged the damage by improving total antioxidant capacity by 1.5 and 2.0 folds, respectively. Moreover, serum MDA level decreased significantly by 20.58 and 51.62 % in fixed and essential oils groups, respectively. The conjugated dienes levels also decreased significantly i.e. 15.98 and 34.35 %, respectively. However, levels of serum MDA and conjugated dienes were higher as compared to normal rats owing to injection of potassium bromate used for induction of oxidative stress [8]. Total antioxidant capacity (TAC) is primarily dependent on antioxidants defence system capabilities and secondly on antioxidants ingested as a part of the diet. Moreover, it also relies on total free radical trapping potential of the body in both normal and oxidative stress conditions. The decreased TAC in oxidative stressed rats can be due to depletion of antioxidants present in the body and increased free radical production. Correlation also provided the evidence that MDA and conjugated dienes were negatively associated with TAC. The investigations conducted by Al-Othman et al. [26] proved that black cumin supplementations hold potential to improve the antioxidant capacity of the body. Antioxidant status i.e. glutathione, α- and γ-tocopherols were improved consequently with experimental diets. Level of glutathione decreased in control by 25.23 %, while increased by 4.30 and 21.82 % in *N. sativa* fixed and essential oils groups, respectively. The restoration of glutathione to near normal ranges is hallmark of the present study. Glutathione along with tocopherols were in linear linkage with antioxidant capacity, while inverse association with that of oxidative damage. These findings further validated our stance that supplementation of diets rich in tocopherols and antioxidant ameliorate the adverse consequences of oxidative stress. Production of free radicals results in lipid peroxidation thus resulting in extensive oxidative damage as indicated by high level of MDA & conjugated dienes. These indicators were modulated in groups of rats consuming fixed and essential oil based diets. According to Alenzi et al. [27], the oils of black cumin and thymoquinone served a protective mechanism against toxicity induced by cyclophosphamide and prevent incidence of necrosis. Formerly, Meral and Kanter [28] observed reduced level of MDA, improved antioxidant capacity and restoration of glutathione because of black cumin supplementation that strengthened the present results.

Lipid peroxides and associated moieties are products of lipid peroxidation that are harmful in progression of diseases such as atherosclerosis and brain damage. However, experimental diets attenuated the damage that certainly be attributed to their rich nutritional profile. In this context, El-Desoky et al. [29] suggested the potential role of tocopherols and nutraceuticals in scavenging free radicals and ameliorating their toxic effects for normal functionality of the body. In another research investigation, Sánchez-Reus et al. [30] reported that oxido-reduction or GSH/GSSG ratio is important in controlling the antioxidant potential of the body. In the present study, NSFO and NSEO modulated the hepatic enzymes activity. The aforementioned results indicated the involvement of complex mechanism in which specialized processes involving glutathione and tocopherols scavenge the oxidants like MDA, lipid peroxides, hydro-peroxides and conjugated dienes. The expressions of hepatic enzymes enhance the antioxidant capacity in the groups of rats fed on experimental diets. In this context, glutathione reductase (GR), glutathione peroxidase (GPx) and glutathione transferase (GST) are important biological catalyst to control peroxides and free radicals production through glutathione cyclic transformation [31].

Role of enzymes in amelioration of oxidative stress is indispensable. Casalino et al. [32] supported the present findings that antioxidant supplementation could avert the hepotocellular damage as a consequences of oxidative stress. The enhanced level of these metabolites triggers cascade of events that results in malfunctioning of the body and even in severe cases death may be the possible penalty. The supplementations of diets with appropriate antioxidant or formulation containing bioactive compounds are useful in reversing the sequential distortion due to free radicals. Some other studies also suggested that active ingredients present in *N. sativa* fixed and essential oils protect the body from nephrotoxicity and hepatotoxicity [4]. *N. sativa* fixed and essential oils ameliorate oxidative stress and maintain body homeostasis by regulating several hematological and serological attributes. The present exploration concluded that *N. sativa* fixed and essential oils have potential to improve the antioxidant status of the body. The diet supplemented with the *N. sativa* oils with special reference to essential oil can act as safeguard against oxidative stress and allied disorders. Future perspectives include testing their effectiveness as nutraceuticals in maladies characterized by oxidative stress like arthritis and osteoporosis.

## Conclusion

Conclusive approach drawn from this efficacy study is, *Nigella sativa* fixed and essential oils hold potential to improve the antioxidant status and decrease antioxidant damage significantly in potassium bromate induced oxidative stressed rats. The oxidative stress and ROS induced negative changes in enzyme expressions but experimental diets modulated hepatic enzymes and immune system including GR, Gpx, GST, MPO, and xanthine oxidase positively. However, there is need to conduct further trials in other types of animal modeling to validate the findings.

## References

[1] Sultan MT, Butt MS, Qayyum MM, Suleria HA (2014). Immunity: plants as effective mediators. Crit Rev Food Sci Nutr.

[2] Andersen CJ, Fernandez ML (2013). Dietary strategies to reduce metabolic syndrome. Rev Endocr Metab Disord.

[3] Sultan MT, Butt MS, Ahmad RS, Pasha I, Ahmad AN, Qayyum MM (2012). Supplementation of *Nigella sativa* fixed and essential oil mediates potassium bromate induced oxidative stress and multiple organ toxicity. Pak J Pharm Sci.

[4] Butt MS, Sultan MT (2010). *Nigella sativa* reduces the risk of various maladies. Crit Rev Food Sci Nutr.

[5] Shabana A, El-Menyar A, Asim M, Al-Azzeh H, Al TH (2013). Cardiovascular benefits of Black Cumin (*Nigella sativa*). Cardiovasc Toxicol.

[6] Zia-Ul-Haq M, Ahmad S, Bukhari SA, Amarowicz R, Ercisli S, Jaafar HZE (2014). Compositional studies and biological activities of some mash bean (*Vigna mungo* (L.) Hepper) cultivars commonly consumed in Pakistan. Biol Res.

[7] Ahmad A, Husain A, Mujeeb M, Khan SA, Najmi AK, Siddique NA, Anwar F (2013). A review on therapeutic potential of *Nigella sativa*: a miracle herb. Asian Pac J Trop Biomed.

[8] Sultan MT, Butt MS, Anjum FM (2009). Safety assessment of *Nigella sativa* fixed and essential oils in normal Sprague Dawley: serological and hematological indices. Food Chem Toxicol.

[9] Uchida K, Satoh T, Ogura Y, Yamaga N, Yamada K (2001). Effect of partial ileal bypass on cholesterol and bile acid metabolism in rats. Yanago Acta Medica.

[10] Ohkawa N, Ohishi J, Yagi K (1979). Assay for lipid peroxides in animal tissues by thiobarbituric acid reaction. Anal Biochem.

[11] Hermes-Lima M, Willmore WG, Storey KB (1995). Quantification of lipid peroxidation in tissue extracts based on Fe(III)xylenol orange complex formation. Free Radic Biol Med.

[12] Corongiu FP, Milla A (1983). An improved method for determining diene conjugation in autoxidized polyunsaturated fatty acids. Chem Biol Interact.

[13] Beutler E (1982). A manual of biochemical methods.

[14] Xu Z, Godber JS (1999). Purification and identification of components of gamma-oryzanol in rice bran oil. J Agric Food Chem.

[15] Sun Y, Oberley LW, Li Y (1988). A simple method for clinical assay of superoxide dismutase. Clin Chem.

[16] Block PP, Karmer R, Paverka M (1980). A simple assay for catalase determination. Cell Biol Monog.

[17] Tappel AL (1978). Glutathione peroxidase and hydroperoxides. Method Enzym.

[18] Paglia DE, Valentine WN (1967). Studies on the quantitative and qualitative characterization of erythrocyte glutathione peroxidase. J Lab Clin Med.

[19] Hillegas LM, Griswold DE, Brickson B, Albrightson-Winslow C (1990). Assessment of myeloperoxidase activity in whole rat kidney. J Pharmacol Meth.

[20] Singh AS, Pal DT, Mandal BC, Singh P, Pathak NN (2002). Studies on changes in some of blood constituents of adult cross-bred cattle fed different levels of extracted rice bran. Pak J Nutr.

[21] Morita O, Tamaki Y, Kirkpatrick JB, Chengelis CP (2008). Safety assessment of heated diacylglycerol oil: Subchronic toxicity study in rats. Food Chem Toxicol.

[22] Wajs A, Bonikowski R, Kalemba D (2008). Composition of essential oil from seeds of *Nigella sativa* L. cultivated in Poland. Flav Frag J.

[23] Khan SH, Ansari J, Abbas G (2012). Black cumin seeds as phytogenic product in broiler diets and its effects on performance blood constituents immunity and caecal microbial population. Ital J Anim Sci.

[24] Wu T, Qi X, Liu Y, Guo J, Zhu R, Chen W, Yu T (2013). Dietary supplementation with purified mulberry (Morus australis Poir) anthocyanins suppresses body weight gain in high-fat diet fed C57BL/6 mice. Food Chem.

[25] Lönn ME, Dennis JM, Stocker R (2012). Actions of ‘antioxidants’ in the protection against atherosclerosis. Free Radic Biol Med.

[26] Al-Othman AM, Ahmad F, Al-Orf SK, Al-Murshed AZ (2006). Effect of dietary supplementation of Ellataria cardamomum and *Nigella sativa* on the toxicity of rancid corn oil in Rats. Int J Pharmacol.

[27] Alenzi FQ, El-Bolkiny YS, Salem ML (2010). Protective effects of *Nigella sativa* oil and thymoquinone against toxicity induced by the anticancer drug cyclophosphamide. Br J Biomed Sci.

[28] Meral I, Kanter M (2003). Effects of *Nigella sativa* L. and Urtica dioica L. on selected mineral status and hematological values in CCl_4_-treated rats. Biol Trace Elem Res.

[29] El-Desoky G, Abdelreheem M, Abdulaziz AL, ALOthman Z, Mahmoud M, Yusuf K (2012). Potential hepatoprotective effects of vitamin E and selenium on hepatotoxicity induced by malathion in rats. Af J Pharm Pharmacol.

[30] Sánchez-Reus MI, del Rio G, Iglesias MA, Elorza I, Slowing M, Benedí K (2007). Standardized Hypericum perforatum reduces oxidative stress and increases gene expression of antioxidant enzymes on rotenone-exposed rats. Neuropharmacology.

[31] Das L, Bhaumik E, Raychaudhuri U, Chakraborty R (2012). Role of nutraceuticals in human health. J Food Sci Technol.

[32] Casalino E, Sblano C, Calzaretti G, Landriscina C (2006). Acute cadmium intoxication induces alpha-class glutathione S-transferase protein synthesis and enzyme activity in rat liver. Toxic.

